# Development and Validation of a Patient-Reported Outcome Scale for Tension-Type Headache

**DOI:** 10.3389/fneur.2021.693553

**Published:** 2021-08-27

**Authors:** Jinke Huang, Weichi Guo, Hui Li, Runsheng Xie, Min Shen, Huimin Wang

**Affiliations:** ^1^The Second Affiliated Hospital of Guangzhou University of Chinese Medicine (Guangdong Provincial Hospital of Chinese Medicine), Guangzhou, China; ^2^Department of Neurology, Shantou Hospital of Traditional Chinese Medicine, Shantou, China; ^3^Department of Standardization of Chinese Medicine, The Second Affiliated Hospital of Guangzhou University of Chinese Medicine (Guangdong Provincial Hospital of Chinese Medicine), Guangzhou, China; ^4^Department of Neurology, Zhejiang Provincial Hospital of Chinese Medicine, Hangzhou, China; ^5^Fuyong People's Hospital of Baoan District, Shenzhen, China

**Keywords:** tension-type headache, patient-reported outcome, classical test theory, reliability, validity

## Abstract

**Objective:** To validate a patient-reported outcome (PRO) measure for patients with tension-type headache (TTH).

**Methods:** Literature analysis, interview, and group discussion were performed to develop an initial TTH-PRO. Thereafter, the initial scale was pre-evaluation in a small range of patients with TTH, and the expert panel made necessary adjustments based on the content feedback. The clinical test was carried out by using the adjusted initial scale. Based on the test results, the items were screened by the method of classical test theory to form the final scale, and the performance evaluation indicators such as validity, reliability, and responsiveness of the final scale were tested.

**Results:** The final formed TTH-PRO scale contained three domains, six dimensions, and 30 items. The split-half reliability, Cronbach's α coefficients, and construct validity of the scale were acceptable, as was feasibility. The responsiveness in the physiological domain was fair, but the overall responsiveness still needed further clinical validation.

**Conclusions:**The TTH-PRO scale has been developed with extensive patient input and demonstrates evidence for reliability and validity. It is complementary to existing evaluation indicators of TTH, emphasizing the patient's experience. Further studies are needed to optimize its items and to verify its clinical applicability for population in more regions and countries.

## Introduction

Tension-type headache (TTH) is the most common primary headache in adults (1). Epidemiological data show that TTH has a global prevalence of 38%, accounting for about 70–80% of all headache patients (2). In the adult population aged 18–65 years, the prevalence rate is between 19.7 and 80% in all regions of the world, TTH has become the second most common chronic disease worldwide (3). TTH can lead to pain and disability, cause suffering for patients, and decrease their quality of life. Furthermore, TTH imposes a serious financial and health service burden on health systems (4). The goal of TTH management is to reduce or terminate headache attacks, prevent headache recurrence, and then improve health-related quality of life.

So far, the clinical evaluation of TTH is mostly limited to the evaluation of the headache condition itself. However, based on the possible relationship between the pathogenesis of TTH and psycho-psychological factors, it is also necessary to comprehensively evaluate the patient's psycho-psychological and social function domains. There is a lack of specific scales for TTH. In order to measure the quality of life of patients, most studies have used general scales such as SF-36 (5); however, this general scale lacks specificity. Therefore, it is necessary to develop disease-specific scales for TTH patients to identify their health status.

Patient-reported outcomes (PROs) are any health status reports that come directly from a patient and generally include domains such as symptoms, functional limitations, psychological, and social domains, which can reflect the health status and the effectiveness of treatment from a patient's perspective. For human diseases, patients are indeed the only source endpoint data for health outcomes, so PROs are increasingly regarded as basic evidence to understand the impact of treatment on patient function and health (6). Meanwhile, PROs can be used to detect individual differences between patients and may be a measure to predict important health outcomes (7). The aim of this study was to develop an understandable, reliable, and valid PRO scale for patients with TTH to facilitate the collection of valuable data from the patient's perspective. This paper reports on the initial item development, item screening, and performance evaluation of the scale.

## Method

**Figure 1:**
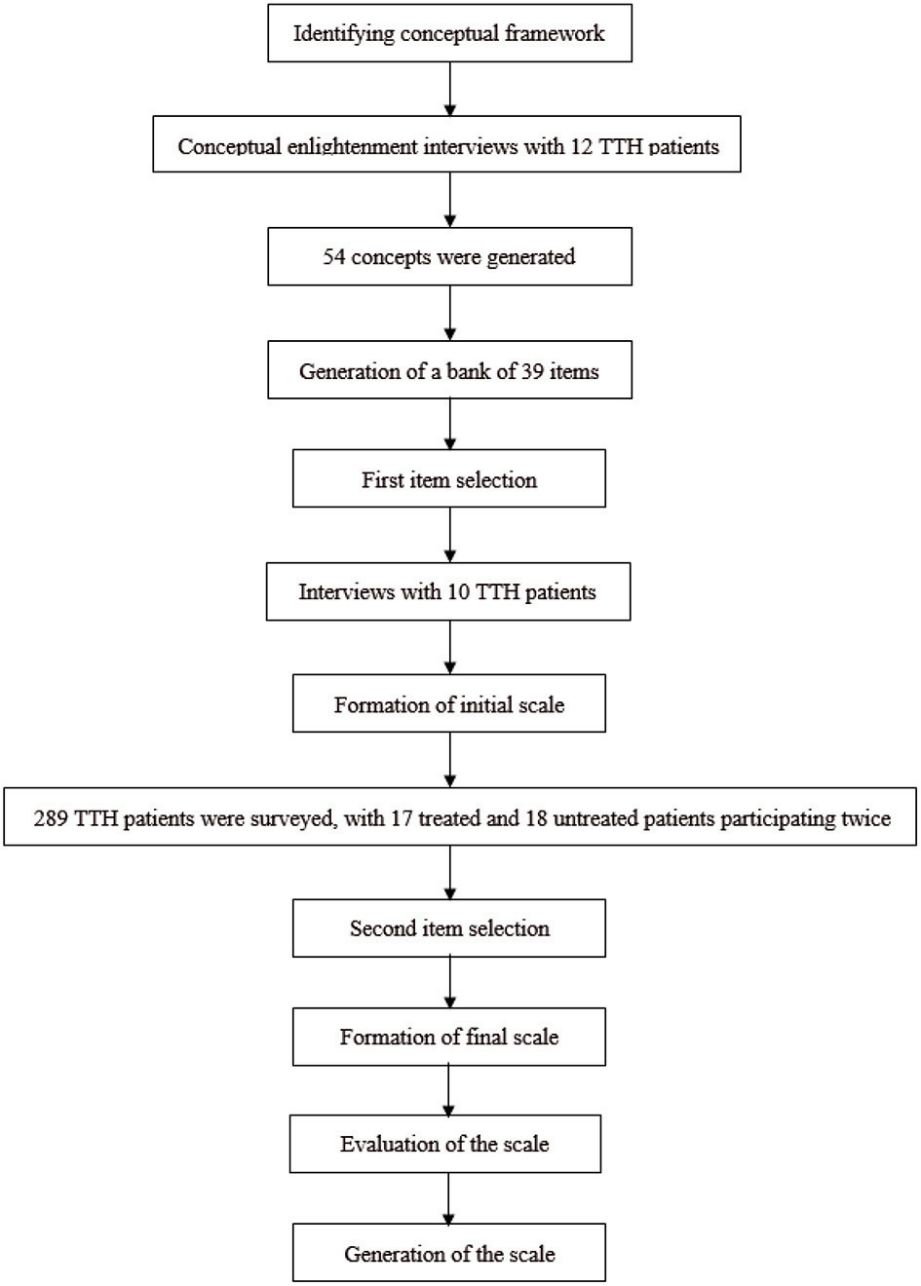
Flowchart of the scale developmental process.

The study protocol was reviewed and approved by the Medical Ethics Committee of Guangdong Provincial Hospital of Chinese Medicine. The TTH-PRO was established in three phases: development of initial scale, initial scale pre-evaluation, and formation and evaluation of final scale. A flowchart of this three-phase developmental process is shown in Figure 1.

### Development of Initial Scale

#### Identifying the Conceptual Framework

The expert panel was composed of neurology clinicians, scale experts, and statistical experts. The conceptual framework was based on theoretical foundations of TTH, literature reference, and input from patients and experts.

#### Building the Item Pool

Using the constructed conceptual framework as a cognitive interview guide, TTH patients attending the outpatient clinic of Guangdong Provincial Hospital of Chinese Medicine were interviewed to freely elaborate on their own symptoms and information concerned during treatment, which was considered to have reached theoretical saturation when patients had no new conceptual output. The interviews were audio-recorded throughout, and the audio-recorded documents were transcribed into text after the interviews, followed by repeated reading of the text materials and three-level coding of the text materials. Based on the coding results, the expert panel held group discussions and formed items for the initial scale.

### Initial Scale Pretest

A small range of patients with TTH in the outpatient clinic of Guangdong Provincial Hospital of Chinese Medicine was tested, and the expert panel made necessary adjustments to the initial scale based on the content feedback during the filling of the scale by the patients. The content of patient feedback after the pre-survey was considered by experts from the following aspects: whether the relevant answer type of the item was appropriate, whether there was a phenomenon of double questioning in the item, whether the item involved personal privacy problems, whether the suggestion was avoided, whether there were difficulties in the understanding of the item, and additional supplements from patients. Language adjustments could be made to the wording of the entries as needed, and items could be deleted or added.

### Development of the Final TTH-PRO and Its Evaluation

#### Field Testing

The initial scale was used to conduct a field survey of TTH patients who visited the neurology clinic of Guangdong Provincial Hospital of Chinese Medicine. During the test, all patients were asked to complete the questionnaire independently on site after receiving a brief introduction from the investigator. Patients with poor vision and difficulty writing were allowed assistance from accompanying families.

#### Methods of Screening Item

Six methods were used to quantitatively optimize the scale items according to the classical measurement theory: discrete trend method, from the perspective of sensitivity to select items; Cronbach's α coefficient method, from the perspective of internal consistency to select items; distribution of item method, from the perspective of central tendency of the answers to the items to select items; correlation coefficient method, from the perspective of representativeness and independence to select items; retest reliability method, from the perspective of stability to select items; and factor analysis method, from the perspective of representative to select items.

#### Validation of the Scale

##### Reliability

The split-half reliability and Cronbach's α coefficient of scale were calculated to assess reliability. For calculating split-half reliability, an odd–even split was adopted. The criterion for acceptable split-half reliability and Cronbach's α was >0.70.

##### Validity

Confirmatory factor analysis with the index of model fit was performed to investigate the factor structure of the scale. The model indicates a good fit when the goodness-of-fit index (GFI), normed fit index (NFI), non-normed fit index (NNFI), incremental fit index (IFI), and comparative fit index (CFI) are all >0.85, and the root mean square residual (RMR) is <0.09. GFI, RMR, NFI, and CFI range from 0 to 1 (8).

##### Responsiveness

Partially treated patients returned with consent to fill out another scale and compare the first and second scores to assess scale responsiveness.

##### Feasibility

In this research, feasibility was evaluated by response rate, completion rate, percentage of missing data, and time required for scale completion.

### Data Analysis Software

Data analyses were conducted using SPSS 26.0, and AMOS 26.0 software.

## Results

### Initial Scale Development Results

#### Conceptual Framework

Based on the theory of TTH, referring to the criteria of clinical evaluation methods related to headache and migraine, and combining with the experience of clinical experts themselves, the members of the expert group conducted a group discussion and concluded that the efficacy evaluation of TTH may involve various aspects, and this framework consists of three domains, including 9 headache-related indicators, a total of 40 physical and psychological related concepts, and 4 function-related problems, for a total of 53 concepts.

#### Item Pool

Twelve patients with TTH were interviewed with concept heuristic; 54 concepts were generated after converting and coding the interview contents. Seventeen secondary categories were collated, namely, headache nature, headache intensity, headache frequency, headache duration, pericranial tenderness, neck discomfort, dizziness, palpitations, nausea, ocular symptoms, sleep status, mental status, anxiety symptoms, depressive symptoms, impaired work ability, impaired activities of daily living, and impaired learning ability. Four main categories were collated, namely, headache, accompanying somatic symptoms, mental/psychological symptoms, and impaired function. Finally, they were aggregated into a core category: PRO scale for TTH. Details could be found in Additional File A.

#### Initial Scale Formation

After repeated discussions, the expert panel concluded that the concepts elicited by the concept heuristic interviews did not exceed the previous conceptual framework. Therefore, the concepts and frameworks formed by the conceptual heuristic interviews were used as the basic compositional source of the scales.

The expert panel repeatedly compressed and integrated these 54 concepts and finally simplified these 54 concepts to generate 39 items. Additionally, the headache dimension and somatic symptom dimension were reintegrated into physiological domain. Finally, the initial scale was developed, which consisted of three domains and 39 items. Details could be found in Additional File B.

After repeated discussion by the expert panel, the response options were measured using a visual analog scale (VAS) ranging from 0 to 10, with a number from “0” to “10” indicating the degree of difference, of which “0” indicates “none”, the degree increases in turn, and “10” indicates “unbearable”.

### Initial Scale Pretest

A pre-survey of 10 patients with TTH attending the outpatient clinic revealed the following issues during the completion of the scale: (a) The item “The degree of normal sexual function” involved privacy, and some patients did not want to fill in it. Considering that it had the possibility of correlation with TTH, it was not removed for the time being. (b) The item “The degree of pain if scalp compression is performed”; some patients complained of headache without conscious compression, and the specific degree of pain was not clear. Considering that most people did not understand the concept of “pericranial tenderness” and had no relevant behavior during headache, it was remarked that the patient may not fill in it if there was no situation in this area; (c) Some patients filled in the three items of “the degree of good mood in the morning”, “the degree of interest in life”, and “the degree of still like what they usually like” as negative items according to inertial thinking; thus, we added a comment prompt.

### Development of the Final TTH-PRO and Its Evaluation

#### Field Testing

From September 2019 to December 2020, a total of 289 patients were included in the survey, of which 17 treated patients and 18 untreated patients were filled in for the second time. Among the included patients, 77 were male and 212 were female; the oldest was 75 years old and the youngest was 13 years old, with an average age of 40.50 ± 14.44 years, mainly young and middle-aged (18–44 years old); the education level was mainly college and undergraduate. Details of the participant characteristics are presented in Table 1.

**Table 1:** Details of the participant characteristics.

		**Number**	**Percent (%)**
Gender	Male	77	26.6
	Female	212	73.4
Age	<18	8	2.8
	18–44	175	60.6
	45–59	70	24.2
	≥60	36	12.5
Education level	Primary school	10	3.5
	Middle School	86	29.8
	College degree or above	186	64.4
	Unclear	7	2.4

#### Item Selection

In this study, six methods were used to quantitatively optimize the scale items according to the classical measurement theory. Items recommended to be deleted by at least three methods were removed. Finally, of these 39 items of the initial scale, 9 items were removed, and the remaining 30 items were retained.

Details could be found in Additional File C.

#### Development of the Final TTH-PRO

Exploratory factor analysis suggested that the construct validity of the initial scale needs to be improved. Therefore, the dimensional structure was further divided on the basis of the original three domains to form the final scale. The final scale consisted of three domains, six dimensions, and 30 items. Details could be found in Table 2 and Additional File D.

**Table 2:** Final scale for tension-type headache.

**Domain**	**Dimension**	**Item**
Physiological	Headache symptom	1. Headache intensity
		2. Number of headache episodes in the last 4 weeks
		3. The location of the headache
		4. Headache nature at the onset of headache
		5. Intensity of scalp pain on scalp compression
	Somatic symptom	6. Degree of neck tightness/soreness
		7. Degree of photophobia/phonophobia, ocular discomfort
		8. Degree of fast heartbeat
		9. Degree of dizziness
		10. Degree of numbness and tingling in hands and feet
		11. Degree of stomach pain, indigestion, constipation
		12. Degree of urinary frequency
Psychological	Negative mood	13. Degree of tension and anxiety
		14. The degree of feeling frightened, irritable, or panicked
		15. The degree to which you feel crazy
		16. Extent of misfortune
		17. How fragile and tired are you
		18. Degree of restlessness or difficulty remaining calm
		19. Degree of difficulty falling asleep, poor sleep, dreaminess, or easy nightmares
		20. How depressed, depressed, and wanting to cry
		21. Degree of irritability
	Negative ideation	22. Extent of hopelessness for future
		23. The extent to which you think you're useless
	Positive emotion	24. The degree to which life is considered enjoyable
		25. I would think that if I died, others would live better
		26. How much you still like what you used to like
Function	Social functioning	27. How much headache affects work
		28. How much headache affects doing housework
		29. How much headache affects interpersonal communication
		30. How much headache affects work affects learning

### Validation of the Scale

#### Reliability

The coefficient of each dimension, the total Cronbach's α coefficient, and the split-half reliability are given in Table 3. As shown, the reliability of this scale was satisfactory.

**Table 3:** Reliability of the scale.

**Domain**	**Cronbach's α**	**Acceptable value**
Physiological	0.674	>0.70
Psychological	0.896	>0.70
Function	0.915	>0.70
Total	0.901	>0.70
Split-half reliability	0.700	>0.70

#### Validity

As shown in Table 4, most of the indexes of fit met the requirements.

**Table 4:** Goodness-of-fit statistic of the scale.

**Indexes**	**χ^2^/df**	**RMSEA**	**GFI**	**AGFI**	**CFI**	**TLI**	**IFI**	**NFI**
Value	1.815	0.053	0.865	0.839	0.904	0.893	0.905	0.811
Acceptable value	>0	>0	>0.85	>0.85	>0.85	>0.85	>0.85	>0.85

#### Responsiveness

A total of 16 treated patients with TTH were included in the clinical survey for the second scale filling in, and the Mann–Whitney *U*-test was used to analyze whether there was a statistically significant difference in the total scores of each domain and the overall scale measured twice before and after treatment. Statistical results revealed that there were no statistically significant (*p* > 0.05) differences in the total scores of psychological domain, functional domain, and global scale between the two measurements. There was a statistically significant (*p* = 0.043) difference in the total score of the physiological domain between the two measurements.

#### Feasibility

Both the completion rate and qualified rate of the TTH-PRO scale were more than 95%. The average completion time was 332 ± 112 s. There were few missing items, with the exception of those about sexual activity. The majority of participants could understand the items.

## Discussion

Because the patient's perspective on the impact of symptoms and functional wellbeing is unique, and some aspects of a condition are known only to the patient, asking patients about their experiences with TTH is essential (9). Additionally, perspectives of physicians and patients are not always consistent. A study comparing physicians' and patients' responses to physical and social functions found that doctors did not recognize or significantly underestimate the functional disabilities reported by patients (10). Therefore, it is necessary to develop a PRO scale for comprehensive TTH outcomes assessment. This study followed the guideline (11) issued by the Food and Drug Administration (FDA) and developed and validated a PRO scale for use in the evaluation of outcomes for TTH patients. To the best of our knowledge, this scale is the first PRO scale on TTH that includes physical, psychological, and social functioning domains.

### Scale Characteristics

Twelve patients with TTH were interviewed with concept heuristic, and 54 concepts were generated after converting and coding the interview contents. These 54 concepts constituted the conceptual framework of the TTH-PRO scale. Based on the conceptual framework developed, the panel held repeated discussions and, in conjunction with feedback on interviews with 10 TTH patients, initially constructed a TTH-PRO scale. The development of the initial scale was mainly based on the results of the qualitative analysis. In order to strive for the characteristics of good representativeness, strong independence and high sensitivity of each item of this scale, 289 TTH patients were investigated using the initial scale. Based on the results of the survey data, six methods were used to quantitatively optimize the scale items according to classical measurement theory. Finally, nine items were removed, and a TTH-PRO scale containing three domains, six dimensions, and 30 items was developed.

Performance validation revealed that the completion rate and qualified rate of the scale were >95%; the average time to complete the scale was 332 ± 112 s and did not exceed a maximum of 20 min, suggesting that most TTH patients were willing to complete the scale survey. Considering the high acceptance and completion rate together with the short completion time, the TTH-PRO scale is feasible for use in clinical practice. From the results of the classical test theory analysis, the coefficient of split-half reliability and almost each dimension was >0.7, and the total Cronbach's α coefficient was >0.9, which demonstrated good reliability of the TTH-PRO scale. The results of the exploratory factor analysis suggested poor construct validity of the scale and, therefore, the scale dimensions were adjusted according to the results of the exploratory factor analysis. Confirmatory factor analysis was used to revalidate the final scale after adjusting the dimension, and the results suggested that almost each index of fit met the ideal requirements. The TTH-PRO measure was multidimensional in nature. Seventeen treated TTH patients provided data for the responsiveness analysis, and the results suggested that there was a statistically significant difference in physical domain, but not in other domains. Since the sample size included in the investigation was too small, the reliability of the analysis results decreased. Additional studies regarding responsiveness validation with patients from multiple centers are required in the future.

### Comparison With Other Questionnaires

Due to the lack of a PRO scale for comprehensive TTH outcomes assessment, current clinical assessment of TTH often refers to the assessment methods of migraine, such as the Migraine Disability Assessment Questionnaire (MIDAS) (12). The MIDAS does cover some of the domains measured specifically for TTH, such as headache frequency, headache intensity, and the effect of headache on work, study, family, and social activities. Furthermore, the Headache Impact Test (HIT-6) (13) is a widely used PRO scale that assesses the negative impact of headaches on normal daily activity. Also, the HIT-6 does cover some of the domains measured specifically for TTH, such as pain, daily activities, headache-related fatigue, irritability, and difficulty concentrating (13). In clinical trials of treatments for TTH, psychosocial distress is an important outcome. However, both MIDAS and HIT-6 are questionnaires that measure the degree of headache-related disability. Psychometric measures, as a concept independent of physical and social functioning distress, are widely overlooked in these two scales. Thus, they are usually used in combination with psychology-related questionnaires in clinical practice to more comprehensively measure the impact of TTH. To measure quality of life in patients with TTH, many studies have used generic questionnaires, such as the SF-36 (14) and the EQ-5D (15). However, such broad questionnaires may not be sensitive to disease-specific symptoms; therefore, development of disease-specific questionnaires for the TTH patients is needed. TTH-specific symptoms are highly subjective and therefore requires self-report. This TTH-PRO scale represents the first TTH-specific PROs to provide a set of scales for patients to report their health status in physical, psychological, and social functioning domains. These concepts are important to clinical trials of treatments for TTH, and they may help to assist patients, physicians, and policymakers in adopting evidence-based treatment decisions relating to treatment benefits and harms (16).

### Limitations and Further Development

This scale has several potential limitations that we will address in future studies. First, the scale appears too complicated. During the development process, 53 concepts were obtained through concept heuristic interviews. Through the first round of qualitative item screening, these 53 concepts were finally reduced into 39 items; the second round of item screening was performed using the quantitative screening method of classical test theory, and 9 items were removed, and the remaining 30 items constituted the final scale. While undergoing two rounds of item screening combining qualitative and quantitative methods, the final scale also seems to be too complicated, and patients may have some difficulties finishing it because of the length of this questionnaire comprising 30 items. The next phase of development and validation of the TTH-PRO will involve the application of quantitative methods to reduce the length of the instrument, such as those based on item response theory. Second, as with all psychometric scale development research, further ongoing validation work is needed. The sample population of this study may not be representative of the entire TTH patient population due to the limited resources (funding and personnel). Almost all TTH patients in this study were from only the Guangdong province in southern China. Thus, further validation of the TTH-PRO should be conducted nationwide with different sociodemographic and clinical characteristics. For example, population-based validation and low or uneducated population data also need to be supplemented. Additionally, a limitation of this study was that the TTH-PRO was developed in China. Thus, additional cultural and/or linguistic validation studies are required if they are to be used in other countries. Finally, the sample of TTH patients was not large enough, as was the number of participants who completed the responsiveness reliability study. A larger sample of TTH patients for establishing responsiveness is needed to more fully generalize the ability of the TTH-PRO to capture true and meaningful changes in patient health during treatment.

## Conclusions

The TTH-PRO scale has been developed with extensive patient input and demonstrates evidence for reliability and validity. This study provides an assessment tool based on PROs for the evaluation of clinical efficacy in TTH. It is complementary to existing evaluation indicators of TTH, emphasizing the patient's experience. Further studies are needed to optimize its items and to verify its clinical applicability for participants in more regions and countries.

## Data Availability Statement

The original contributions presented in the study are included in the article/Supplementary Material, further inquiries can be directed to the corresponding author.

## Ethics Statement

The study protocol was reviewed and approved by the Medical Ethics Committee of Guangdong Provincial Hospital of Chinese Medicine.

## Author Contributions

JH, WG, and HL: study concept, design, and acquisition of data. JH, WG, HL, and RX: analysis and interpretation of data. JH and WG: drafting of manuscript. All authors critical revision of manuscript for important intellectual content.

## Conflict of Interest

The authors declare that the research was conducted in the absence of any commercial or financial relationships that could be construed as a potential conflict of interest.

## Publisher's Note

All claims expressed in this article are solely those of the authors and do not necessarily represent those of their affiliated organizations, or those of the publisher, the editors and the reviewers. Any product that may be evaluated in this article, or claim that may be made by its manufacturer, is not guaranteed or endorsed by the publisher.

## Abbreviations

TTH: tension-type headache
PRO: patient-reported outcome
FDA: Food and Drug Administration
MIDAS: Migraine Disability Assessment Questionnaire.
