# Supplementary material for: Development and Validation of a Patient-Reported Outcome Scale for Tension-Type Headache
Source: Front Neurol. 2021 Aug 27;12:693553. doi: 10.3389/fneur.2021.693553 (PMC8430245; doi:10.3389/fneur.2021.693553)
Supplement: Supplementary file 1 [file Data_Sheet_1.doc]

Additional file A. Three-level coding results of concept-inspired interviews

| Concept | Number of mentions | Secondary Category | Main Category | Core Category |
| --- | --- | --- | --- | --- |
| Pressure | 1 | Headache nature | Headache | PRO measures for TTH |
| Vibration sense | 1 |
| Tightness | 2 |
| Unspeakable oddness | 1 |
| Bloating sensation | 1 |
| Soreness | 1 |
| Numbness | 1 |
| Distension | 1 |
| Tingling sensation | 1 |
| Girdle sensation | 1 |
| Reduction of headache intensity | 2 | Headache intensity |
| Strong headache | 1 |
| Reduced seizure frequency | 1 | Frequency of headache |
| Long duration | 1 | Headache duration |
| Duration Decreased | 2 |
| Headache site tenderness | 1 | Peri-cranial tenderness |
| Tenderness | 1 |
| Neck tightness | 1 | Neck discomfort | Somatic symptom |
| Drowsiness | 3 | Dizziness |
| Dizziness | 1 |
| Rapid heartbeat | 1 | Palpitations |
| Feeling sick | 1 | Nausea |
| Photophobia | 1 | Ocular Symptoms |
| Eye soreness | 1 |
| Upwind tearing | 1 |
| Eye fatigue | 1 |
| Blurred vision | 1 |
| Poor quality sleep | 6 | Sleep condition | Mental/Psychological |
| Sleep improvement | 1 |
| Insomnia | 1 |
| Trouble sleeping | 1 |
| Dreaminess | 2 |
| Want to sleep | 2 |
| Mental stress | 1 | Mental status |
| Mental instability | 1 |
| No spirit | 1 |
| Poor mental state | 1 |
| Mental stress | 2 |
| Outburst of temper | 2 | Anxiety symptoms |
| Hysteria | 1 |
| Feeling anxious | 4 |
| Irritability | 3 |
| Cardiac palsy | 1 |
| Fear | 1 | Depressive symptoms |
| Depressed mood | 2 |
| Poor mood | 1 |
| Affects work | 6 | Impaired working function | Function impaired |
| Affects life | 1 | Activities of daily living impaired |
| Want to lie down | 1 |
| Exhaustion | 3 |
| Bradyphrenia | 1 | Learning impairment |
| Affects attention | 1 |
| Affects reading time | 1 |
| Don't want to learn | 1 |

Additional file B. Initial scale for tension-type headache

| Field | Items |
| --- | --- |
| Physiological | 1. Headache intensity |
|  | 2. Number of headache episodes in the last 4 weeks |
|  | 3. The location of the headache |
|  | 4. Headache nature at the onset of headache |
|  | 5. Duration of headache episodes |
|  | 6. Intensity of scalp pain on scalp compression |
|  | 7. Degree of neck tightness/ soreness |
|  | 8. Degree of nausea and vomiting |
|  | 9. Degree of photophobia/ phonophobia, ocular discomfort |
|  | 10. Degree of fast heartbeat |
|  | 11. Degree of dizziness |
|  | 12. Degree of dyspnea |
|  | 13. Degree of numbness and tingling in hands and feet |
|  | 14. Degree of stomach pain, indigestion, constipation |
|  | 15. Degree of urinary frequency |
|  | 16. The degree to which the hand is prone to sweating |
|  | 17. Degree of blushing and fever |
|  | 18. The degree of trembling hands and feet |
| Psychological | 19. Degree of tension and anxiety |
|  | 20. The degree of feeling frightened, irritable, or panicked |
|  | 21. The degree to which you feel crazy |
|  | 22. Extent of misfortune |
|  | 23. How fragile and tired are you |
|  | 24. Degree of restlessness or difficulty remaining calm |
|  | 25. The degree of difficulty falling asleep, poor sleep, dreaminess, or easy nightmares |
|  | 26. How depressed, depressed, and wanting to cry |
|  | 27. Degree of good mood in the morning |
|  | 28. Extent of hopelessness for the future |
|  | 29. Degree of irritability |
|  | 30. The extent to which you think you're useless |
|  | 31. The degree to which life is considered enjoyable |
|  | 32. I would think that if I died, others would live better |
|  | 33. How much you still like what you used to like |
| Function | 34. The degree of difficulty in doing things or making decisions |
|  | 35. Normal degree of sexual function |
|  | 36. How much headache affects work |
|  | 37. How much headache affects doing housework |
|  | 38. How much headache affects interpersonal communication |
|  | 39. How much headache affects work affects learning |

Additional file C. Summary of the item selection

| Item | Discrete trend | Cronbach's α coefficient | Distribution of item | Correlation coefficient | Retest reliability | Factor analysis | Enrollment number | Selected items* |
| --- | --- | --- | --- | --- | --- | --- | --- | --- |
| 1 | × |  |  | × |  |  | 4 | √ |
| 2 |  | × |  | × |  |  | 4 | √ |
| 3 |  | × |  | × |  |  | 4 | √ |
| 4 | × |  |  | × |  |  | 4 | √ |
| 5 |  | × |  | × |  | × | 3 | × |
| 6 |  |  |  | × |  |  | 5 | √ |
| 7 |  |  |  | × |  | × | 4 | √ |
| 8 |  |  |  | × | × | × | 3 | × |
| 9 |  |  |  | × |  | × | 4 | √ |
| 10 |  |  |  | × |  |  | 5 | √ |
| 11 |  |  |  | × |  |  | 5 | √ |
| 12 | × |  | × | × |  |  | 3 | × |
| 13 |  |  |  | × |  | × | 4 | √ |
| 14 |  |  |  | × |  |  | 5 | √ |
| 15 |  |  | × | × |  |  | 4 | √ |
| 16 | × |  | × | × |  |  | 3 | × |
| 17 | × |  | × | × | × |  | 2 | × |
| 18 | × |  | × | × |  |  | 3 | × |
| 19 |  |  |  |  |  |  | 6 | √ |
| 20 |  |  |  |  |  |  | 6 | √ |
| 21 |  |  |  |  |  |  | 6 | √ |
| 22 |  |  |  |  |  |  | 6 | √ |
| 23 |  |  |  |  |  |  | 6 | √ |
| 24 |  |  |  |  |  |  | 6 | √ |
| 25 |  |  |  | × |  |  | 5 | √ |
| 26 |  |  |  |  |  |  | 6 | √ |
| 27 |  | × |  | × |  |  | 4 | √ |
| 28 |  |  | × | × |  |  | 4 | √ |
| 29 |  |  |  |  |  |  | 6 | √ |
| 30 |  |  | × |  |  |  | 5 | √ |
| 31 |  |  |  |  |  |  | 6 | √ |
| 32 | × |  | × | × | × |  | 2 | × |
| 33 |  |  |  | × | × |  | 4 | √ |
| 34 |  | × | × | × |  | × | 2 | × |
| 36 |  |  |  |  |  |  | 6 | √ |
| 37 |  |  |  |  |  |  | 6 | √ |
| 38 |  |  |  |  |  |  | 6 | √ |
| 39 |  |  |  |  |  |  | 6 | √ |

×: Items recommended to be deleted; √: Items recommended to be retained; ‘*’: Items recommended to be deleted by at least three methods, were finally removed.

Additional file D.

**A Patient Reported Outcome Scale for Tension- Type Headache**

| **Headache symptom** | | | | | | | |
| --- | --- | --- | --- | --- | --- | --- | --- |
| 1. Headache intensity? | | | | | | | |
| |  |  |  |  |  |  |  |  |  |  | | --- | --- | --- | --- | --- | --- | --- | --- | --- | --- |   0 1 2 3 4 5 6 7 8 9 10 | | | | | | | |
| 2. The number of headache episodes in the last 4 weeks is times. | | | | | | | |
|  | | | | | | | |
| 3. The location of the headache? | | | | | | | |
| 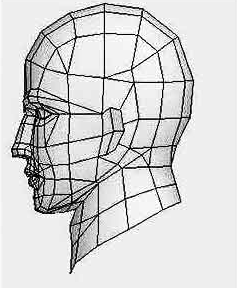 | 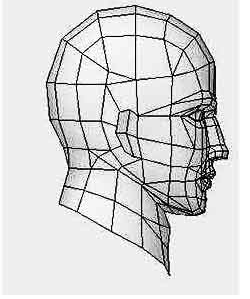 | | 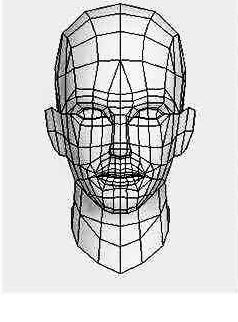 | | | 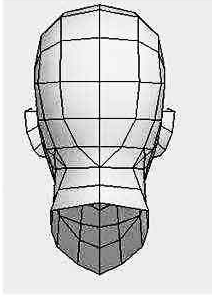 | |
| 4. Headache nature at the onset of headache? | | | | | | | |
| □Pulsatile □Hoop/compression/girdle sensation □Lightning sensation □Explosive sensation □Stinging □Swelling pain □Soreness □Numbness □Undescribed □Others | | | | | | | |
| 5. Intensity of scalp pain on scalp compression? | | | | | | | |
| |  |  |  |  |  |  |  |  |  |  | | --- | --- | --- | --- | --- | --- | --- | --- | --- | --- |   0 1 2 3 4 5 6 7 8 9 10    **Somatic symptom** | | | | | | | |
| 6. Degree of neck tightness? | |  | |  |  |  |  |
| |  |  |  |  |  |  |  |  |  |  | | --- | --- | --- | --- | --- | --- | --- | --- | --- | --- |   0 1 2 3 4 5 6 7 8 9 10 | | | | | | | |
| 7. Degree of photophobia/ phonophobia, ocular discomfort? | | | | | | | |
| |  |  |  |  |  |  |  |  |  |  | | --- | --- | --- | --- | --- | --- | --- | --- | --- | --- |   0 1 2 3 4 5 6 7 8 9 10 | | | | | | | |
| 8. Degree of fast heartbeat? | | | | | | | |
| |  |  |  |  |  |  |  |  |  |  | | --- | --- | --- | --- | --- | --- | --- | --- | --- | --- |   0 1 2 3 4 5 6 7 8 9 10 | | | | | | | |
| 9. Degree of dizziness? | | | | | | | |
| |  |  |  |  |  |  |  |  |  |  | | --- | --- | --- | --- | --- | --- | --- | --- | --- | --- |   0 1 2 3 4 5 6 7 8 9 10 | | | | | | | |
| 10. Degree of numbness and tingling in hands and feet? | | | | | | | |
| |  |  |  |  |  |  |  |  |  |  | | --- | --- | --- | --- | --- | --- | --- | --- | --- | --- |   0 1 2 3 4 5 6 7 8 9 10 | | | | | | | |
| 11. Degree of stomach pain, indigestion, constipation? | | | | | | | |
| |  |  |  |  |  |  |  |  |  |  | | --- | --- | --- | --- | --- | --- | --- | --- | --- | --- |   0 1 2 3 4 5 6 7 8 9 10 | | | | | | | |
| 12. Degree of urinary frequency? | | | | | | | |
| |  |  |  |  |  |  |  |  |  |  | | --- | --- | --- | --- | --- | --- | --- | --- | --- | --- |   0 1 2 3 4 5 6 7 8 9 10 | | | | | | | |
| **Negative mood** | | | | | | | |
| 13. Degree of tension and anxiety? | | | | | | | |
| |  |  |  |  |  |  |  |  |  |  | | --- | --- | --- | --- | --- | --- | --- | --- | --- | --- |   0 1 2 3 4 5 6 7 8 9 10 | | | | | | | |
| 14. The degree of feeling frightened, irritable, or panicked? | | | | | | | |
| |  |  |  |  |  |  |  |  |  |  | | --- | --- | --- | --- | --- | --- | --- | --- | --- | --- |   0 1 2 3 4 5 6 7 8 9 10 | | | | | | | |
| 15. The degree to which you feel crazy? | | | | | | | |
| |  |  |  |  |  |  |  |  |  |  | | --- | --- | --- | --- | --- | --- | --- | --- | --- | --- |   0 1 2 3 4 5 6 7 8 9 10 | | | | | | | |
| 16. Extent of misfortune? | | | | | | | |
| |  |  |  |  |  |  |  |  |  |  | | --- | --- | --- | --- | --- | --- | --- | --- | --- | --- |   0 1 2 3 4 5 6 7 8 9 10 | | | | | | | |
| 17. How fragile and tired are you? | | | | | | | |
| |  |  |  |  |  |  |  |  |  |  | | --- | --- | --- | --- | --- | --- | --- | --- | --- | --- |   0 1 2 3 4 5 6 7 8 9 10 | | | | | | | |
| 18. Degree of restlessness or difficulty remaining calm? | | | | | | | |
| |  |  |  |  |  |  |  |  |  |  | | --- | --- | --- | --- | --- | --- | --- | --- | --- | --- |   0 1 2 3 4 5 6 7 8 9 10 | | | | | | | |
| 19. Degree of difficulty falling asleep, poor sleep, dreaminess, or easy nightmares? | | | | | | | |
| |  |  |  |  |  |  |  |  |  |  | | --- | --- | --- | --- | --- | --- | --- | --- | --- | --- |   0 1 2 3 4 5 6 7 8 9 10 | | | | | | | |
| 20. How depressed, depressed, and wanting to cry? | | | | | | | |
| |  |  |  |  |  |  |  |  |  |  | | --- | --- | --- | --- | --- | --- | --- | --- | --- | --- |   0 1 2 3 4 5 6 7 8 9 10 | | | | | | | |
| 21. Degree of irritability? | | | | | | | |
| |  |  |  |  |  |  |  |  |  |  | | --- | --- | --- | --- | --- | --- | --- | --- | --- | --- |   0 1 2 3 4 5 6 7 8 9 10 | | | | | | | |
| **Negative ideation**  22. Extent of hopelessness for future? | | | | | | | |
| |  |  |  |  |  |  |  |  |  |  | | --- | --- | --- | --- | --- | --- | --- | --- | --- | --- |   0 1 2 3 4 5 6 7 8 9 10 | | | | | | | |
| 23. The extent to which you think you're useless? | | | | | | | |
| |  |  |  |  |  |  |  |  |  |  | | --- | --- | --- | --- | --- | --- | --- | --- | --- | --- |   0 1 2 3 4 5 6 7 8 9 10 | | | | | | | |
| **Positive emotion**  24. The degree to which life is considered enjoyable? | | | | | | | |
| |  |  |  |  |  |  |  |  |  |  | | --- | --- | --- | --- | --- | --- | --- | --- | --- | --- |   0 1 2 3 4 5 6 7 8 9 10 | | | | | | | |
| 25. I would think that if I died, others would live better? | | | | | | | |
| |  |  |  |  |  |  |  |  |  |  | | --- | --- | --- | --- | --- | --- | --- | --- | --- | --- |   0 1 2 3 4 5 6 7 8 9 10 | | | | | | | |
| 26. How much you still like what you used to like? | | | | | | | |
| |  |  |  |  |  |  |  |  |  |  | | --- | --- | --- | --- | --- | --- | --- | --- | --- | --- |   0 1 2 3 4 5 6 7 8 9 10 | | | | | | | |
| **Social functioning** | | | | | | | |
| 27. How much headache affects work? | | | | | | | |
| |  |  |  |  |  |  |  |  |  |  | | --- | --- | --- | --- | --- | --- | --- | --- | --- | --- |   0 1 2 3 4 5 6 7 8 9 10 | | | | | | | |
| 28. How much headache affects doing housework? | | | | | | | |
| |  |  |  |  |  |  |  |  |  |  | | --- | --- | --- | --- | --- | --- | --- | --- | --- | --- |   0 1 2 3 4 5 6 7 8 9 10 | | | | | | | |
| 29. How much headache affects interpersonal communication? | | | | | | | |
| |  |  |  |  |  |  |  |  |  |  | | --- | --- | --- | --- | --- | --- | --- | --- | --- | --- |   0 1 2 3 4 5 6 7 8 9 10 | | | | | | | |
| 30. How much headache affects work affects learning? | | | | | | | |
| |  |  |  |  |  |  |  |  |  |  | | --- | --- | --- | --- | --- | --- | --- | --- | --- | --- |   0 1 2 3 4 5 6 7 8 9 10 | | | | | | | |
